# Chronic stress negatively impacts wound healing, welfare, and stress regulation in internally tagged Atlantic salmon (*Salmo salar*)

**DOI:** 10.3389/fphys.2023.1147235

**Published:** 2023-04-03

**Authors:** Miiro Ilmari Virtanen, Monica Fengsrud Brinchmann, Deepti Manjari Patel, Martin Haugmo Iversen

**Affiliations:** Faculty of Biosciences and Aquaculture, Nord University, Bodø, Nordland, Norway

**Keywords:** chronic stress, wound healing, inflammation, welfare, tagging

## Abstract

The desire to understand fish welfare better has led to the development of live monitoring sensor tags embedded within individuals for long periods. Improving and understanding welfare must not come at the cost of impaired welfare due to a tag’s presence and implantation process. When welfare is compromised, the individual will experience negative emotions such as fear, pain, and distress, impacting the stress response. In this study, Atlantic salmon (*Salmo salar*) underwent surgical implantation of a dummy tag. Additionally, half of this group was introduced to daily crowding stress. Both groups and an untagged group were followed for 8 weeks using triplicate tanks per group. Sampling took place once a week, and where stress was given, it was conducted 24 h before sampling. Stress-related measurements were taken to understand if tagging caused chronic stress and explore the chronic stress response and its impact on wound healing. Primary stress response hormones measured included CRH, dopamine, adrenocorticotropic hormone, and cortisol. Secondary stress response parameters measured included glucose, lactate, magnesium, calcium, chloride, and osmolality. Tertiary stress response parameters measured included weight, length, and five fins for fin erosion. Wound healing was calculated by taking the incision length and width, the inflammation length and width, and the inside wound length and width. The wound healing process showed that stressed fish have a larger and longer-lasting inflammation period and a slower wound healing process, as seen from the inside wound. The tagging of Atlantic salmon did not cause chronic stress. In contrast, daily stress led to an allostatic overload type two response. ACTH was elevated in the plasma after 4 weeks, and cortisol followed elevation after 6 weeks, highlighting a breakdown of the stress regulation. Fin erosion was elevated alongside cortisol increase in the stressed group. This data suggests that tagging previously unstressed fish in a controlled environment does not negatively affect welfare regarding stress responses. It also indicates that stress delays wound healing and increases the inflammatory response, highlighting how continued stress causes a breakdown in some stress responses. Ultimately, the tagging of Atlantic salmon can be successful under certain conditions where proper healing is observed, tag retention is high, and chronic stress is not present, which could allow for the possible measurement of welfare indicators *via* smart-tags.

## 1 Introduction

**FIGURE 1:**
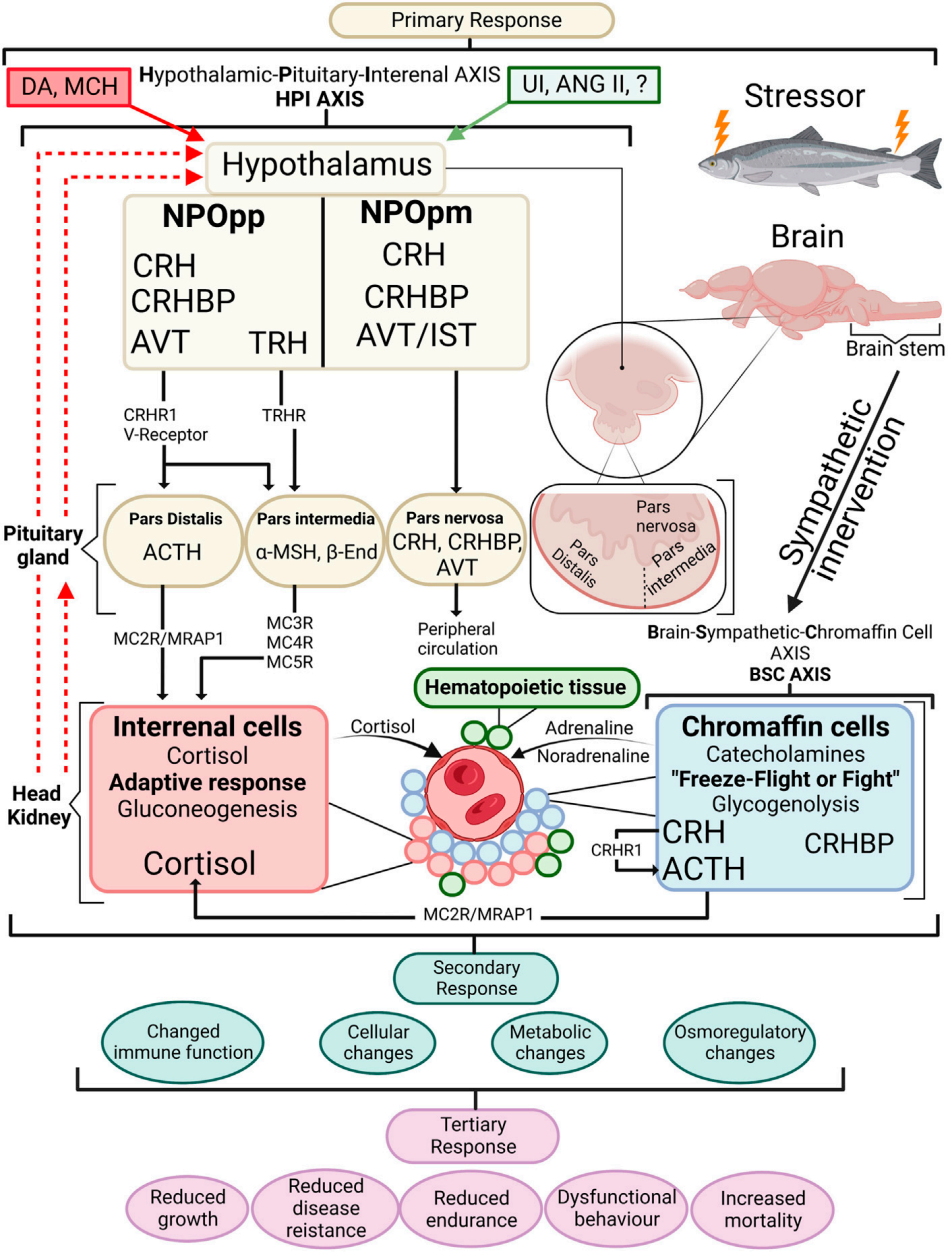
An overview of the stress response in fish with emphasis on the endocrine response. When a stressor is perceived, the primary stress response is activated by the BSC axis in the chromaffin cells of the head kidney where the initial freeze-fight or flight response releases adrenalin and noradrenalin in the circulation. Along with chromaffin cells the head kidney contains haematopoeitic tissue and interrenal cells. The HPI axis is activated starting within the hypothalamus where cortisol releasing hormone (CRH) containing axons are sent from the nucleus preopticus (NPOpp) to cells of adrenocorticotropic hormone (ACTH) within the pituitary. Simultaneously CRF-binding protein (CRFBP) is brought to the ACTH cells where CRF/CRFBP decides CRF bioactivity. CRF/CRFBP along with thyrotropin-releasing hormone (TRH) control the α-melanophore stimulating hormone (α-MSH) which has a role in stimulating cortisol release from the interrenal cells. ACTH through the melanocortin 2 receptor (MC2R)-melanocortin 2 accessory protein 1 (MRAP1) complex induces cortisol synthesis at the interrenal. Negative feedback is visible by dashed red lines showing negative feedback on the pituitary gland and hypothalamus. Stimulatory effects (green box) produced include urocortin I (UI) angiotensin II (ANG II) as well as arginine vasotocin (AVT) and isotocin (IST) among others, and negative feedforwards (red box) include dopamine (DA) and melanin-concentrating hormone (MCH). Primary stress responses lead to secondary stress responses which are physiological adjustments to a stressor. Tertiary responses are whole-animal responses and occur after secondary responses take place. β-End (beta-endorphin). Redrawn and modified from Gorissen and Flik (2016) and Khansari et al. (2017a).

Global salmon production in 2019 reached 3.8 million tons worldwide; on average, 15% of the salmon produced is lost, which is of significant concern for the producer, government, and the public (Ellis et al., 2012; Bang Jensen et al., 2020). The push to explain the underlying cause of mortality has been accompanied by the increasing need to understand and document fish welfare (Noble et al., 2018; Oliveira et al., 2021). Fish welfare has no universally defined definition or way of measurement. Still, one common consensus is to use The Farm Animal Welfare Committee (FAWC) guidelines for the Five Freedoms, which represent a framework of animal welfare, and to follow three types of welfare approaches; function-based, nature-based, and feelings-based (FAWC, 1996; Ashley, 2007; Kristiansen and Bracke, 2020). Regardless of how animal welfare is defined, one standard agreement can be made that it is the quality of life felt from the eyes of the animal itself that ultimately must be considered (Stien et al., 2013; Noble et al., 2018). To measure fish welfare, one integral component associated closely is the stress response, which can have both an improving or malicious effect on the wellbeing and survival of the individual, depending on whether the nature of stress is either adaptive or maladaptive (Wendelaar Bonga, 2011). The concept of stress introduced by Selye (1950) has been altered and modified over the years, and in recent years the notion of allostasis has been submitted to complement the concept of stress. Thus, more precisely describing the intricate role of primary mediators (e.g., glucocorticosteroids) (McEwen, 1998; McEwen and Wingfield, 2003; Iversen and Eliassen, 2014). Figure 1 summarises the complexity of the stress response in fish, focusing on the endocrine response.

While measurements of stress and welfare offer insight into the wellbeing of aquatic fish, the need how to measure them has led to the adoption of new methods. One method uses innovative tagging technology to provide real-time data on farmed fish’s environment, behaviour, and physiology (Macaulay et al., 2021). Tagging gives fish a possible voice to communicate behavioural and physiological responses within a population through the tags that analyse and interpret multiple forms of data. Tagging technology has been implemented in terrestrial animal agriculture for real-time monitoring of individual animals to improve the response to compromised animal welfare in an approach termed; “precision livestock farming” (PLF) (Berckmans, 2014). The number of individuals farmed in aquaculture and the hardships of studying behaviour in the aquatic environment present a challenging task for farmers. Some of the issues that have been associated with decreased welfare and increased mortality are; sea lice infestation, diseases, water quality, temperature, salinity, predators, and algal blooms, (Ellis et al., 2012; Bang Jensen et al., 2020; Hvas et al., 2021; Oliveira et al., 2021). Tagging offers a possible tool for understanding these welfare challenges. However, tagging every individual within a grow-out pen at sea becomes economically and logistically unrealistic; thus, using a percentage of individual fish to represent the whole population is advisable (Føre et al., 2018). The invasive nature of tagging is a paradox, for, in the context of aquatic welfare, one should not compromise welfare when assessing it. As such, the implantation and presence of the tag cannot influence the expected behaviour, physiology, and welfare of individuals for them to be considered a viable representative of the population (Alfonse et al., 2020; Macaulay et al., 2021). Thus, it is essential to build scientific knowledge on the interplay between the invasive tagging process and any adverse welfare effects.

The process of tagging with larger internal tags requires an incision, which leads to the formation of an open wound. The wound must heal normally under stressful aquaculture conditions for precision livestock farming to be considered viable in aquaculture. Previous studies on salmonid wound healing show that the healing cascade consists of the immediate re-epithelialization of the wound coinciding with a longer than 2-week inflammation period (Sveen et al., 2019). Further tissue repair and remodelling can last several months, while scale regeneration can take over a year when the underlying muscle is damaged, even though the skin pigmentation resembles pre-wounding (Fontenot and Neiffer, 2004; Schmidt et al., 2016). This wound-healing cascade for humans has been shown to slow down significantly with stress (Christian et al., 2006). In Atlantic salmon, high fish density delays the epidermal and dermal repair of the wound site (Sveen et al., 2019). Surgical tagging, however, creates a manipulated wound site consisting of sutures and a deep wound that penetrates through the individual, which has seen limited research considering the effects on fish welfare. Therefore, the outcomes of tagging in aquaculture require more transparency, as outlined by Macaulay et al. (2021). Clear and consistent reporting of results will allow faster governmental and industrial adoption of tagging to ensure better welfare during the tagging process and provide unaffected welfare data from tagged individuals, ultimately providing live welfare status to farmers.

This study aims to document and explore how surgically tagged fish under unstressed and stressed conditions affect their wound-healing ability and fish welfare. Aquaculture practices consist of fish held at high stocking density to increase yield and profit. Thus, daily crowding stress was utilised as a chronic stress condition. Atlantic salmon (*Salmo salar*) was chosen due to its importance in aquaculture and its significance as the most researched farmed fish species with behaviour monitoring tags (Macaulay et al., 2021).

## 2 Materials and methods

The Norwegian Food Safety Authority approved the experiment on 24.05.2019 and it is registered under FOTS ID 19447.

### 2.1 Fish and housing

The 225 (270 total, including unsampled) Atlantic salmon sampled to conduct the study were of the strain AquaGen QTL-Innova SHIELD, hatched on 08.12.2018 at Cermaq hatchery department in Hopen, Norway. The fish arrived at the Mørkvedbukta research station (Nord University; Bodø 67.2804^◦^ N, 14.4049^◦^ E) on 09.05.2019, where they were smoltified and then transferred (21.04.2020) to nine isolated off-white circular indoor tanks (30 fish per tank) (1.0 m^−3^) with a continuous flow of seawater with salinity 33.5‰, the temperature of 7.3 ± 0.3°C, and oxygen level of 97.7% ± 5.2%. The fish were kept under a 24-h light regime, with dry feed dispensed automatically in excess (Supreme, Skretting AS). The acclimation lasted 68 days until the start of the experiment on 28.06.2020. 30 fish with a mean weight of 1.01 ± 0.3 kg and a mean length of 43.1 ± 3.3 cm were held in nine tanks (in triplicate) at the start of the experiment.

### 2.2 Experimental design

**FIGURE 2:**
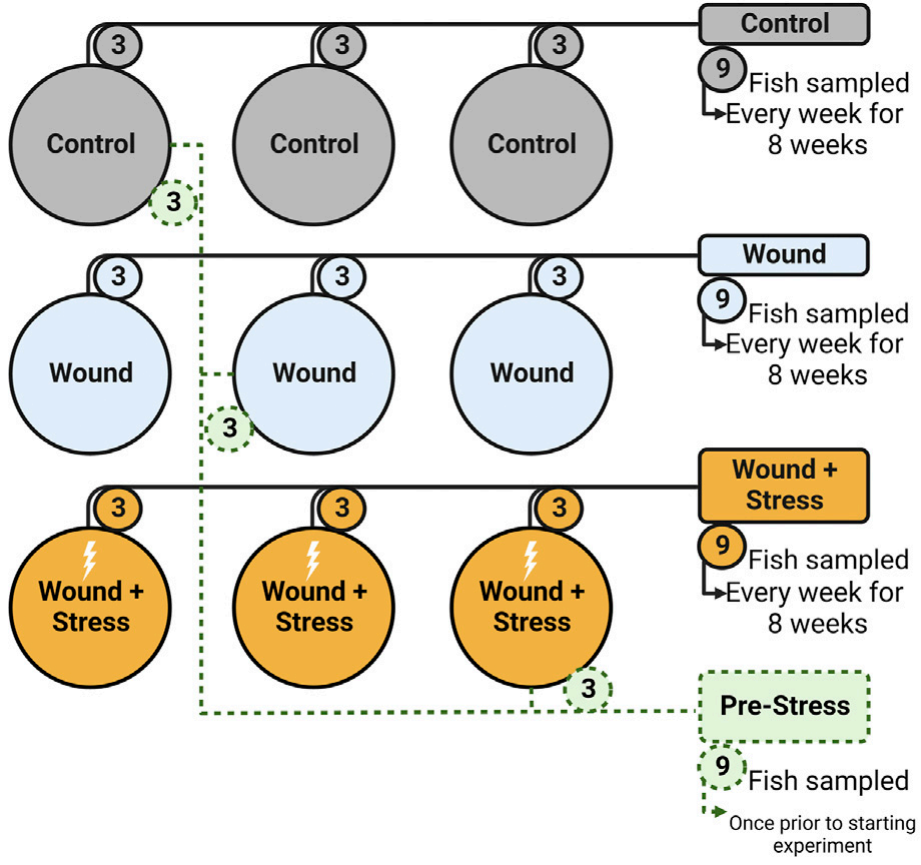
Experimental setup showing the nine tanks, three groups (Control, Wound, Wound + Stress), and the pre-Stress group. Numbers represent how many fish were taken from each tank on a sampling day (n = 9).

To determine the effect of chronic stress and the impact of a wound after tagging, three experimental groups (in triplicate, a total of nine tanks) were used: (1) Control, (2) Wound, and (3) Wound + Stress. Control groups were undisturbed healthy individuals (Figure 2). The wound group had a dummy tag surgically implanted according to Section 2.2.2 and was used to show the effect of wound healing on unstressed fish. The wound + stress group underwent the same surgical procedure as the wound group but was also exposed to a daily crowding stressor to study the effect of chronic stress on stress responses, wound healing, and welfare. The experiment lasted 8 weeks, and sampling was conducted once per week on each experimental group for 8 weeks. Additional sampling was done 1 day before the start of the experiment, considered the pre-stress group.

#### 2.2.1 Chronic stress

The experimental group “Wound + Stress” was subject to daily crowding stress. Stress was achieved by lowering the water level in the tank (triplicate) until only half the fish’s body was submerged. The water was allowed to be at its lowest level for 30 s, and then the tank was filled up to normal levels. From when the water started to drain out of the tanks to when the water was back to normal took approximately 30 min (±30 s). The total fish density changed abruptly from 30 kg/m^3^ to 315 kg/m^3^ in these 30 min. The crowding stress was applied daily (starting on day one) at random times during an 8-h window. The last stress would always be used 24 h before sampling. Earlier studies by Iversen and Eliassen (2014) have shown that this crowding stressor was enough to elicit chronic stress after 4 weeks of exposure.

#### 2.2.2 Surgical implantation

The implantation of a dummy smart tag with size 0.4 × 2 cm made with a 3D printer using high-density polyethene provided by Artic Seafood Group was placed into the fish in the wound group and the wound + stress group on day 0 of the experiment. All the fish in one tank were moved to a holding tank from which two fish at a time were moved into a small tank containing a dose of 60 mg/L of Finquel vet (MSD Animal Health Norge AS), with an aerator and a water pump. Once the fish were under the effect of general anaesthesia (stage 4), as described by Iversen et al. (2003), they were placed on the surgery table. The water pump connected to the anaesthesia bath continuously pumped the same 60 mg/L of Finquel vet water over the gills while the procedure took place. An incision of 1.5 cm was made with a scalpel (No 23, Swann-Morton, Sheffield, United Kingdom) and a tool that only allowed the blade to move 1.5 cm on the ventral surface, located between the pectoral fins and 1.5 cm behind the base of the fins. The dummy tag was cleaned with 75% ethanol and dried before inserting it into the abdominal cavity, where it would be next to the pyloric caeca. Two stitches were sewn with superficial interrupted knots tied on opposite sides of the incision using a non-absorbable 4/0 monofilament suture (www.resorba.com). Sutures were made to be sewn through the muscle not to affect the internal wound surface. The operation took between 60–90 s; once operated, the individual would be placed into a wake-up bath before putting it into its main tank, where it would be held for the duration of the experiment. Miiro Virtanen did all surgical procedures to avoid differing surgeon effects.

### 2.3 Sampling

Sampling occurred once per week for 8 weeks for each experimental group. Three fish from each tank belonging to the same group were randomly taken and placed in anaesthetic 10 L bucket baths containing Finquel vet (120 mg/L). One by one, fish were sampled for blood and then euthanised by cutting their spinal cord near the brain with precise scalpel placement. The fish was then measured for its weight, length, fin scores, and all wound parameters. Two blood samples were taken from the caudal vein complex with a 3 mL heparinised syringe. The blood was measured for glucose and lactate and placed in 1.5 mL Eppendorf tubes (VWR, Norway). It was then centrifuged at 5,000 rpm for 5 min. After this, the plasma was removed into new Eppendorf tubes and stored at −40°C pending further analysis. 72 fish were sampled for each experimental group, totalling 216 fish plus the nine pre-stress fish for the whole experiment.

### 2.4 Measurements and analytical methods

#### 2.4.1 Plasma cortisol

Plasma cortisol levels were measured using the Enzyme-Linked Immunosorbent Assay (ELISA) method, using a DRG Cortisol ELISA kit (EIA-1887, DRG Instruments GmbH, Germany, 2020). The antibody-coated 96-well microplate provided works based on the principle of competitive binding. The manufacturer’s instructions were followed, and the absorbance of each well was read by a 450 nm microtiter plate reader (Tecan Sunrise Remote, Bergman diagnostics, Austria) and corrected for optical imperfections by subtracting from 540 nm. Standards were run in triplicates, while samples, negative control, and positive control (also used to determine plate-to-plate variation) were run in duplicates. The assay has a dynamic range between 1.3–800 ng/mL. The intra- and interassay coefficients were 8.1% and 7.7%, respectively (EIA-1887, DRG Instruments GmbH, Germany, 2020). Plasma cortisol is expressed in nmol/L (nM).

#### 2.4.2 Plasma ACTH

Plasma adrenocorticotropic hormone (ACTH) levels were measured by utilising the Enzyme-Linked Immunosorbent Assay (ELISA) method, using a Cusabio ACTH ELISA kit adapted for fish (CSB- E15926Fh, Cusabio Houston, TX, United States). The antibody-coated 96-well microplate provided works based on the principle of competitive binding. The manufacturer’s instructions were followed, and the absorbance of each well was read by a 450 nm microtiter plate reader (Tecan Sunrise Remote, Bergman diagnostics, Austria) and corrected for optical imperfections by subtracting from 540 nm. Standards were run in triplicates, while samples, negative control, and positive control (also used to determine plate-to-plate variation) were run in duplicates. The assay has a dynamic range between 75–1,200 pg/mL. The intra- and interassay coefficients were <15% and <15%, respectively (CSB-E15926Fh, Cusabio Houston, TX, United States). Plasma ACTH is expressed in pmol/L (pM).

#### 2.4.3 Plasma CRH

Plasma corticotropin-releasing hormone (CRH) levels were measured by utilising ELISA method, using an Abebio CRH ELISA kit adapted for fish (AE64596FI, Wuhan Abebio science, Wuhan, China). The antibody-coated 96-well microplate provided works based on the principle of competitive binding. The manufacturer’s instructions were followed, and the absorbance of each well was read by a 450 nm microtiter plate reader (Tecan Sunrise Remote, Bergman diagnostics, Austria) and corrected for optical imperfections by subtracting from 540 nm. Standards were run in triplicates, while samples, negative control, and positive control (also used to determine plate-to-plate variation) were run in duplicates. The assay has a dynamic range between 0.8–20 ng/mL. The intra- and interassay coefficients were <8% and <10%, respectively (AE64596FI, Wuhan Abebio science, Wuhan, China). Plasma CRH is expressed in ng/mL.

#### 2.4.4 Plasma dopamine

Dopamine (DA) levels were measured by ELISA method, using a Cusabio DA ELISA kit adapted for fish (CSB-EQ027496FI, Cusabio Houston, TX, United States). The antibody-coated 96-well microplate provided works based on the principle of competitive binding. The manufacturer’s instructions were followed, and the absorbance of each well was read by a 450 nm microtiter plate reader (Tecan Sunrise Remote, Bergman diagnostics, Austria) and corrected for optical imperfections by subtracting from 540 nm. Standards were run in triplicates, while samples, negative control, and positive control (also used to determine plate-to-plate variation) were run in duplicates. The assay has a dynamic range between 62.5–1,000 pg/mL. The intra- and interassay coefficients were <15% and <15%, respectively (CSB-EQ027496FI, Cusabio Houston, TX, United States). Plasma dopamine is expressed in pg/mL.

#### 2.4.5 Plasma ions

Plasma was analysed for ions using the analytical instrument Respons 910 (DiaSys, Holzheim, Germany) with 1:2 sample dilution in ion-free water. Ions included for analysis were; chloride (Cl−, Chloride 21 FS, 40–170 mM), magnesium (Mg^2+^, Magnesium XL FS, 0.08–3.00 mM) and calcium (Ca^2+^, Calcium P FS, 0.22–4.00 mM). The interassay coefficient between the Response 910 analysis and the previously described analysis by Iversen and Eliassen (2014) for fish using 20 samples regarding chlorine and magnesium showed a variation of 8.3% and 2.1%, respectively.

#### 2.4.6 Plasma osmolality

Plasma osmolality was analysed using a Fiske One-Ten Osmometer (Fiske Associates, Norwood, MA, United States).

#### 2.4.7 Blood glucose

Blood glucose concentrations were measured from whole blood using the handheld Freestyle Freedom Lite^TM^ (Abbott Diabetes Care Inc., United Kingdom) and test strips from Ascensia Diabetes Care. Whole blood was applied to the test strips immediately after sampling. Glucose concentrations were read between 1.1–27.8 mmol/L (mM). Values below the detection limit were set to 1.1 mM (lower range limit).

#### 2.4.8 Blood lactate

Blood lactate concentrations were measured using the handheld Lactate Scout + ^TM^ with its test strips (EKF Diagnostics for life). Whole blood was applied to the test strips immediately after sampling. Lactate concentrations were read between 0.5–25 mmol/L (mM). Values below the detection limit were set to 0.5 mM (lower range limit).

#### 2.4.9 Fin erosion

Visual scores were given for five fins of the fish. These included the pectoral, pelvic, dorsal, anal, and caudal fins. Fin scores were given based on a compressed version of a scoring system introduced by (Hoyle et al., 2007) with minor modifications utilising an ordinal scale of 0, 1, 2, and 3, corresponding to erosion (0% of fin eroded), mild erosion (1%–24% eroded), moderate (25%–49% of fin eroded) and severe erosion (>50% of fin eroded), respectively. To reduce subjective variation, the scoring of fin erosion was done by Miiro Virtanen, and fish was provided randomly and blindly throughout the different experimental groups by another person.

#### 2.4.10 Wound parameters

**FIGURE 3:**
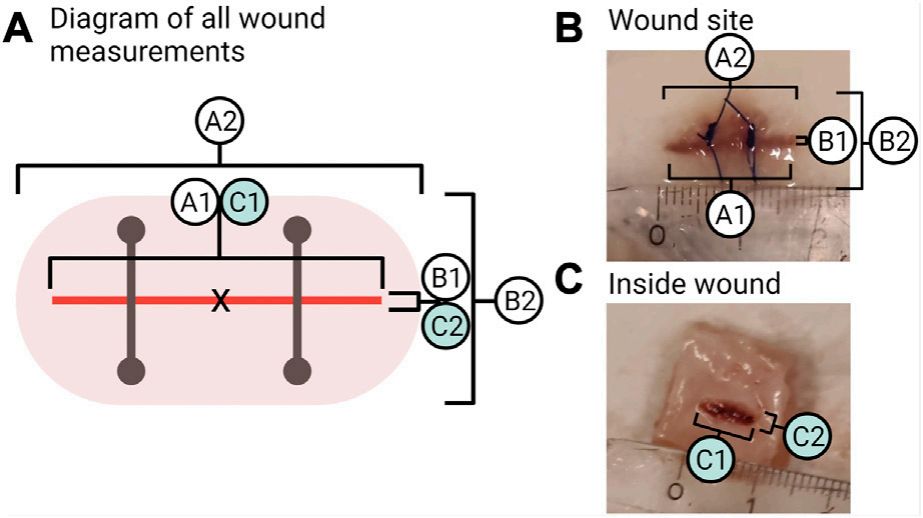
Wound measurements. **(A)** Shows all points measured of the wound where A1 is the length of the incision and B1 is the width of the incision. A2 is the length of inflammation, and B2 is the width of inflammation. C1 is the inside wound length, and C2 is the inside wound width. **(B)** Photo of a sample from week 2 in the group “wound + stress” showing the measurements of the outside wound for A1, B1, and A2, B2. **(C)** Photo of a sample from week 2 in the group “wound + stress” showing the inside measurements for C1 and C2.

To measure visible wound healing, six points of measurements of the wound region, as seen in Figure 3, were taken (to the nearest millimetre) from each fish during sampling. The incision length and width were measured (A1 and B1; Figure 3). The wound inflammation (red area) was measured by taking the length and width of inflammation (A2 and B2; Figure 3) from the incision point to the maximum point where inflammation can be seen. Inflammation is not static; the place and distribution between the sides of the incision will vary as the cause of inflammation can be due to the wound, the suture, abrasion, or all combined. Due to this and the fact that inflammation was random in its orientation, an oval shape of the mean width and length of inflammation was used to create an area showing the mean distribution of inflammation around the incision. The centre point is marked X in Figure 3A, where the measurements cross-section pass. Inside wound healing was measured by taking the length and width of the visible wound (C1 and C2; Figure 3). As the injury was a narrow incision, the length for A1 was measured as the visual line. Thus, even if healed, the line would still be measured as the visible disruption of the skin. While for the width of the incision (B1), a healed wound was determined as the presence of no measurable open wound. The inflammation length (A2) was measured until it matched the length of the incision (A1), after which the reported inflammation would be the same as the incision length.

### 2.5 Statistical analysis

All data were tested for normality using Shapiro-Wilk’s test and for homogeneity of variance using Levene’s test. One-way ANOVA was performed from the start of the experiment (pre-stress) to each sampling time point and within each sampling point for each physiological and morphological parameter measured to identify the difference within groups and the pre-stress. Tukey’s *post hoc* multiple comparisons test was carried out to determine if the F-values were significant. When data did not follow Gaussian distribution, Kruskal–Wallis ANOVA (non-parametric) with Dunn’s multiple comparisons test was conducted. To study the relationship between plasma cortisol (primary stress response) and fin erosion (tertiary stress response), one performed a Spearman’s rank correlation coefficient (rho or r). Wound measurements, inflammation area and inside wound area were compared using an unpaired *t*-test with Welch’s correction, and if not following normal distribution unpaired Mann-Whitney test was done. The triplicate tanks were compared for all parameters with the method previously described for group analysis to identify any tank effect. No such tank effects were discovered. Statistical analysis and graphs were performed and created with GraphPad PRISM v9.3.1 (GraphPad Software, Inc., California, United States). The significance of the results was determined at a *p* < 0.05. Results are represented as mean with standard deviation (SD). Statistical significance in figures and tables within a group at different times compared to pre-stress was indicated by *, and the difference between the experimental groups at the same sampling time was indicated by superscripts a and b in tables and graphs at a significance level of 0.05.

## 3 Results

During the experimental period, mortality and tag retention was recorded. The control group’s survival rate was 98.6%, while a survival rate of 97.2% and tag retention of 100% were registered for the wound group, and a 98.6% survival rate and 100% tag retention were recorded for the wound + stress group. There were no significant differences between the groups.

### 3.1 Primary stress responses

The primary stress responses measured include CRH, dopamine, ACTH, and osmolality, as shown in Table 1.

**TABLE 1:** Mean ± SD (n = 9) for CRH (ng/mL), Dopamine (DA) (pg/mL), ACTH (pmol/L) and cortisol (nmol/L) in control (C), wound (W) and wound + stress (WS) during an 8-week experimental timespan (primary stress response).

	Group	Pre-stress	Week 1	Week 2	Week 3	Week 4	Week 5	Week 6	Week 7	Week 8
		*M ± SD*	*M ± SD*	*M ± SD*	*M ± SD*	*M ± SD*	*M ± SD*	*M ± SD*	*M ± SD*	*M ± SD*
CRH (ng/mL)	C	1.48 ± 0.29	1.73 ± 0.51	1.75 ± 0.53	1.51 ± 0.36^a^	1.60 ± 0.43	1.36 ± 0.57	2.49 ± 0.86*^a^	3.13 ± 2.46*^a^	3.70 ± 3.18*
	W		1.62 ± 0.24	1.77 ± 0.25	2.01 ± 1.09^ab^	1.65 ± 0.41	1.78 ± 0.77	1.65 ± 0.56^ab^	1.91 ± 1.91^b^	3.46 ± 3.41
	WS		1.78 ± 0.28	1.78 ± 0.28	5.44 ± 6.10*^b^	2.71 ± 1.63*	1.83 ± 0.99	1.28 ± 0.44^b^	1.71 ± 0.66^ab^	2.56 ± 1.34

DA (pg/mL)	C	243.4 ± 18.0	292.2 ± 62.7^ab^	267.5 ± 41.0	278.3 ± 26.4*	292.7 ± 61.4	303.5 ± 48.0*^ab^	329.0 ± 48.8*^ab^	301.5 ± 21.1*^a^	427.1 ± 109.8*
	W		302.6 ± 39.8*^a^	293.5 ± 22.5*	285.6 ± 21.0*	305.3 ± 57.4*	324.7 ± 37.7*^a^	347.9 ± 30.9*^a^	365.7 ± 68.8*^b^	391.8 ± 80.2*
	WS		253.7 ± 45.5^b^	272.8 ± 45.9	281.4 ± 26.2*	277.8 ± 33.4	282.0 ± 16.5*^b^	304.1 ± 17.2*^b^	300.2 ± 22.6*^a^	385.6 ± 49.0*

ACTH (pmol/L)	C	34.46 ± 4.14	44.83 ± 3.69*	39.28 ± 9.35	41.99 ± 6.48	52.84 ± 8.50*^a^	55.93 ± 12.37*^a^	47.63 ± 9.32^a^	55.22 ± 14.01^a^	50.35 ± 4.38^a^
	W		44.6 ± 6.91*	45.53 ± 7.98*	43.73 ± 10.10	52.94 ± 9.04*^a^	47.99 ± 6.13^a^	76.64 ± 29.00*^b^	83.42 ± 36.66*^ab^	62.48 ± 12.84*^ab^
	WS		39.93 ± 6.66	51.09 ± 9.30*	48.53 ± 4.35*	198.95 ± 81.55*^b^	124.39 ± 78.72*^b^	79.31 ± 6.10*^b^	132.5 ± 98.76*^b^	72.33 ± 25.40*^b^

Cortisol (nM)	C	15.12 ± 14.87	18.66 ± 38.77	16.91 ± 28.29	9.74 ± 16.21	9.50 ± 14.02	17.14 ± 19.64	3.46 ± 3.60^a^	1.68 ± 0.00^a^	1.68 ± 0.00^a^
	W		4.54 ± 7.08	5.08 ± 10.19*	11.26 ± 28.75	8.97 ± 18.12	5.46 ± 11.11	9.54 ± 15.08^a^	20.67 ± 30.37^a^	10.52 ± 14.60^a^
	WS		31.92 ± 40.60	5.07 ± 10.15*	11.19 ± 28.53	39.44 ± 69.90	40.14 ± 43.61	80.89 ± 36.81*^b^	71.51 ± 62.77^b^	93.38 ± 98.58*^b^

C = Control, W = Wound, WS = Wound and Stress. Values represent means ± SD, n = 9 per treatment/week. Means in a column (week) within the same measurement that have differing superscript letters a-b indicate significant differences (*p* < 0.05). Asterisks* show a significant difference compared to the Pre-stress group (*p* < 0.05).

#### 3.1.1 CRH

In week 3, the average plasma CRH in the wound + stress group was significantly higher than in the control and wound groups (*p* = 0.0033). The control group’s plasma CRH was significantly higher than the wound group at week 7 (*p* = 0.016), and the wound + stress group at week 6 (*p* = 0.0012). Significant differences in the pre-stress group were observed in the wound + stress group at weeks 3 and 4 and in the control groups at weeks 6, 7, and 8 (Table 1).

#### 3.1.2 Dopamine

The changes in levels of plasma DA during the experiment are shown in Table 1. The average plasma DA in the wound group was significantly higher compared to the wound + stress group during weeks 1 (*p* = 0.027), 5 (*p* = 0.049), 6 (*p* = 0.019), and 7 (*p* = 0.011). The average plasma DA in the wound group was significantly higher compared to the control group in week 7 (*p* = 0.012). Significant differences compared to the pre-stress group were observed in the control group at weeks 3, 5, 6, 7, and 8, at all-time points for the wound group, and during weeks 3, 5, 6, 7, and 8 for the wound + stress group (Table 1).

#### 3.1.3 ACTH

**FIGURE 4:**
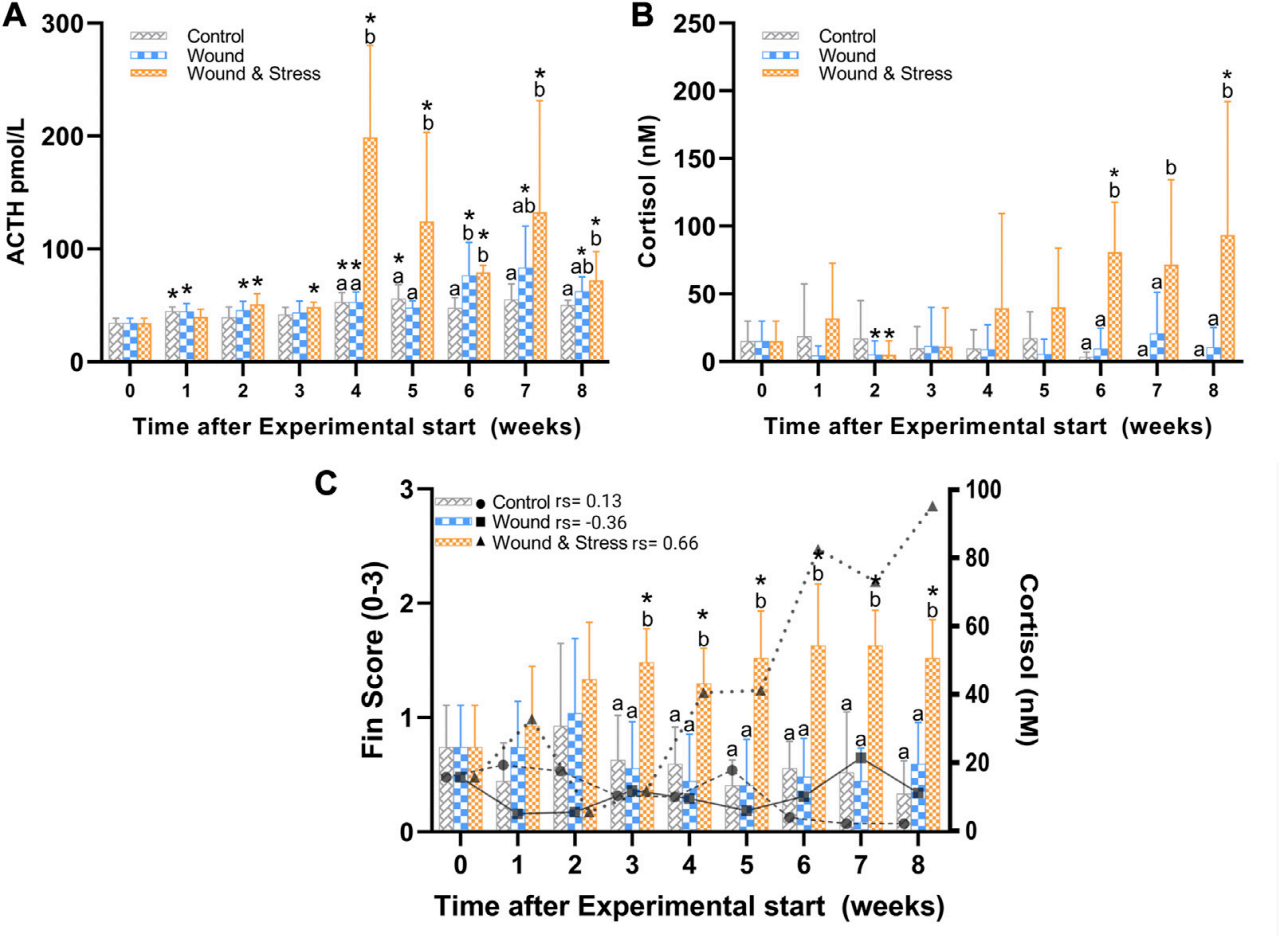
The average (±SD) changes in plasma ACTH **(A)**, cortisol **(B)**, and a combined average of pelvic, pectoral, and anal fin erosion scores with an overlay of cortisol **(C)** in the control, wound, and wound + stress group (n = 9) during an 8-week experimental time. * Indicates a significant difference (*p* < 0.05) from each group to the pre-stress value and letters a-b represent the significant difference (*p* < 0.05) within a single time point for all three groups where sharing letters means no significance between the groups. In **(C)** the individual rho or rs is represented in terms of a Spearman’s rank correlation coefficient indicated next to the group legend.

The changes in levels of plasma ACTH during the experiment are shown in Figure 4. The average plasma ACTH in the wound + stress group was significantly higher than in the control group during weeks 4 (*p* = 0.0007), 5 (*p* = 0.012), and 7 (*p* = 0.009). Additionally, the wound + stress group had significantly higher average plasma ACTH than the wound group during weeks 4 (*p* = 0.001) and 5 (*p* = 0.005). Significant differences compared to the pre-stress group were observed in the control group at weeks 1, 4, and 5, while for the wound group, it was observed during weeks 1, 2, 4, 6, 7, and 8, and for the wound + stress group during all weeks after week 1(Table 1).

#### 3.1.4 Cortisol

The changes in plasma cortisol levels during the experiment are shown in Figure 4. The average plasma cortisol in the wound + stress group was significantly higher than in the control group during weeks 6 (*p* = 0.0005), 7 (*p* = 0.0001), and 8 (*p* < 0.0001), and for the wound group during weeks 6 (*p* = 0.0001), 7 (*p* = 0.0037), and 8 (*p* = 0.0026). The stress + wound group also had significantly higher cortisol levels in weeks 6 and 8 than pre-stress values. The wound and stress + wound groups had significantly lower cortisol levels in week 2 compared to the pre-stress values (Table 1).

### 3.2 Secondary stress responses

The secondary stress responses measured include glucose, lactate, magnesium, calcium, chloride, and osmolality, as shown in Table 2.

**TABLE 2:** Mean ± SD (n = 9) of glucose (mM), lactate (mM), magnesium (mM), calcium (mM) and osmolality (mOsm/kg) in control (C), wound (W) and wound + stress (WS) during an 8-week experimental timespan (secondary stress responses).

	Group	Pre-stress		Week 1	Week 2	Week 3	Week 4	Week 5	Week 6	Week 7	Week 8
		*M ± SD*	*M ± SD*	*M ± SD*	*M ± SD*	*M ± SD*	*M ± SD*	*M ± SD*	*M ± SD*	*M ± SD*
Glucose (mM)	C	2.71 ± 0.13		3.66 ± 0.38*	3.78 ± 0.82*	3.43 ± 0.37*	3.32 ± 0.47*	3.52 ± 0.39*	3.49 ± 0.22*	3.58 ± 0.39*	3.68 ± 0.43*
	W			4.07 ± 0.72*	3.32 ± 0.32*	3.44 ± 0.34*	4.06 ± 1.14*	3.27 ± 0.30*	3.63 ± 0.43*	3.57 ± 0.48*	3.48 ± 0.16*
	WS			4.03 ± 0.84*	3.54 ± 0.49*	3.58 ± 0.32*	3.44 ± 0.38*	3.43 ± 0.30*	3.64 ± 0.26*	3.69 ± 0.64*	3.82 ± 0.41*

Lactate (mM)	C	3.23 ± 0.69		3.67 ± 0.97	3.71 ± 1.02	3.99 ± 0.74	4.60 ± 0.71*	4.39 ± 0.97*	4.62 ± 1.09*	4.79 ± 0.81*	5.73 ± 0.90*^a^
	W			4.56 ± 1.87	3.71 ± 1.17	5.08 ± 1.35*	4.34 ± 1.05*	3.72 ± 0.65	5.12 ± 2.04*	5.14 ± 1.45*	4.27 ± 0.65*^b^
	WS			4.33 ± 1.31	4.20 ± 1.29	4.89 ± 1.53*	4.14 ± 0.51*	3.41 ± 0.58	5.29 ± 1.27*	4.54 ± 1.57	4.87 ± 0.99*^ab^

Magnesium (mM)	C	1.20 ± 0.35		1.18 ± 0.17	1.43 ± 0.41^a^	1.12 ± 0.28	1.02 ± 0.24	1.05 ± 0.13	0.96 ± 0.31^a^	1.45 ± 0.47	1.44 ± 0.38^ab^
	W			1.41 ± 0.35	1.01 ± 0.23^b^	1.29 ± 0.34	1.17 ± 0.28	1.14 ± 0.28	1.60 ± 0.30*^b^	1.50 ± 0.33	1.75 ± 0.25*^a^
	WS			1.34 ± 0.49	1.07 ± 0.16^b^	1.40 ± 0.36	1.14 ± 0.20	1.06 ± 0.20	1.40 ± 0.41*^b^	1.22 ± 0.43	1.17 ± 0.34^b^

Calcium (mM)	C	3.17 ± 0.29		3.42 ± 0.25	3.49 ± 0.36	3.29 ± 0.20	3.24 ± 0.37	3.31 ± 0.15	2.97 ± 0.65^a^	3.54 ± 0.27	3.53 ± 0.30*^a^
	W			3.32 ± 0.36	3.19 ± 0.39	3.40 ± 0.31	3.29 ± 0.25	3.23 ± 0.43	3.61 ± 0.19^b^	3.49 ± 0.26	3.76 ± 0.22*^a^
	WS			3.22 ± 0.33	3.19 ± 0.21	3.46 ± 0.23	3.33 ± 0.24	3.32 ± 0.17	3.39 ± 0.25^ab^	3.10 ± 0.59	3.18 ± 0.35*^b^

Chloride (mM)	C	133.1 ± 5.9		128.1 ± 8.7*^a^	131.4 ± 20.8	115.4 ± 13.2*^a^	120.4 ± 12.3*^ab^	106.9 10.2*^a^	117.9 ± 11.5*	112.8 ± 7.0*	108.9 ± 12.4*^a^
	W			108.8 ± 10.7*^b^	116.8 ± 10.4	103.8 ± 12.3*^a^	109.3 ± 12.8*^a^	114.7 ± 13.5*^a^	119.9 ± 14.4	119.6 ± 7.0*	116.4 ± 15.3*^a^
	WS			126.8 ± 11.2*^a^	125.2 ± 10.1	130.9 ± 10.2*^b^	131.3 ± 14.0*^b^	128.4 ± 6.4^b^	125.8 ± 12.1	117.6 ± 13.8*	131.3 ± 6.7^b^

Osmolality (mOsm/Kg)	C	408.3 ± 25.7		348.8 ± 10.3*^a^	381.3 ± 41.6	403.3 ± 37.9	416.6 ± 30.1	429.7 ± 30.9	433.9 ± 38.0	448.1 ± 39.3*	437.8 ± 27.1^a^
	W			389.1 ± 28.7^b^	399.7 ± 40.2	427.6 ± 37.1	417.6 ± 30.7	441.8 ± 81.0	466.7 ± 56.3*	432.0 ± 26.5	418.6 ± 29.0^ab^
	WS			393.9 ± 32.2^b^	402.3 ± 28.1	400.0 ± 30.7	406.4 ± 30.9	398.2 ± 29.9	435.7 ± 34.3	413.8 ± 32.2	403.2 ± 21.8^b^

C = Control, W = Wound, WS = Wound and Stress. Values represent means ± SD, n = 9 per treatment/week. Means in a column (week) within the same measurement that have differing superscript letters a-b indicate significant differences (*p* < 0.05). Asterisks* show a significant difference compared to the Pre-stress group (*p* < 0.05).

#### 3.2.1 Glucose

Blood glucose had no difference between the three groups at any time point, while all groups and time points were significantly higher than pre-stress values (Table 2).

#### 3.2.2 Lactate

Blood lactate was higher only during week 8 (*p* = 0.0046) in the control group compared to the wound group. Plasma lactate differed from the pre-stress levels in the control group during weeks 4, 5, 6, 7, and 8, for the wound group during weeks 3, 4, 6, 7, and 8, and for the wound + stress group during weeks 3, 4, 6, and 8 (Table 2).

#### 3.2.3 Magnesium

The average plasma magnesium in the control group was significantly higher at week 2 compared to the wound (*p* = 0.014) and wound + stress group (*p* = 0.035). The wound group (*p* = 0.0017) and wound + stress group (*p* = 0.036) were significantly higher at week 6 compared to the control group. Additionally, the wound group had significantly higher magnesium values in week 8 (*p* = 0.0089) compared to the wound + stress group. Plasma magnesium differed from the pre-stress values for the wound group during weeks 6 and 8 (Table 2).

#### 3.2.4 Calcium

The average plasma calcium in the control group was significantly lower at week 6 (*p* = 0.0086) compared to the wound group and significantly higher at week 8 (*p* = 0.046) compared to the wound + stress group. Additionally, the wound group had significantly higher average plasma calcium values in week 8 (*p* = 0.0008) than the wound + stress group. Significant differences compared to the pre-stress group were observed in all groups only at week 8 (Table 2).

#### 3.2.5 Chloride

The average plasma chloride in the control group was significantly higher at week 1 (*p* = 0.0015) compared to the wound group and significantly lower at week 3 (*p* = 0.028), 5 (*p* = 0.0006), and 8 (*p* = 0.0016) compared to the wound + stress group. Additionally, the wound group had significantly lower average plasma osmolality values in week 1 (*p* = 0.003), 3 (*p* = 0.0002), 4 (*p* = 0.0041), 5 (*p* = 0.027), and 8 (*p* = 0.037) compared to the wound + stress group. Significant differences compared to the pre-stress group were observed in the control group at weeks 1, 3, 4, 5, 6, 7, and 8, at weeks 1, 3, 4, 5, 7, and 8 for the wound group and during weeks 1, 3, 4, and 7 for the wound + stress group (Table 2).

#### 3.2.6 Osmolality

The average plasma osmolality in the control group was significantly lower at week 1 compared to the wound group (*p* = 0.0074) and wound + stress group (*p* = 0.0028) and significantly higher compared to the wound + stress group at week 8 (*p* = 0.026). Significant differences compared to the pre-stress group were observed in the control group at weeks 1 and 7 and week 6 for the wound group. In contrast, no differences were found in the pre-stress and wound + stress groups (Table 2).

### 3.3 Tertiary stress responses

The growth is shown in Table 3. A significant decrease in weight was observed for the wound group compared to the control group for week 8 (*p* = 0.035). Additionally, the control group showed a significant increase in length compared to pre-stress values at weeks 7 and 8. At week 8, the control group was significantly longer than the wound and wound and stress group.

**TABLE 3:** Mean ± SD (n = 9) of weight (kg), length (cm), pectoral, pelvic, anal, caudal, and dorsal fins (scored 0–3) in control (C), wound (W), and wound + stress (WS) during an 8-week experimental timespan (tertiary stress responses).

	Group	Pre-stress	Week 1	Week 2	Week 3	Week 4	Week 5	Week 6	Week 7	Week 8
		*M ± SD*	*M ± SD*	*M ± SD*	*M ± SD*	*M ± SD*	*M ± SD*	*M ± SD*	*M ± SD*	*M ± SD*
Weight (kg)	C	1.01 ± 0.27	0.96 ± 0.26	0.98 ± 0.28	1.20 ± 0.33	1.10 ± 0.22	1.20 ± 0.17	1.32 ± 0.31*	1.54 ± 0.22*	1.67 ± 0.43*^a^
	W		0.97 ± 0.25	1.07 ± 0.30	1.09 ± 0.31	1.07 ± 0.11	1.25 ± 0.32	1.32 ± 0.30*	1.42 ± 0.23*	1.24 ± 0.27^b^
	WS		1.16 ± 0.31	0.87 ± 0.21	1.14 ± 0.22	1.15 ± 0.26	1.25 ± 0.39	1.31 ± 0.23	1.38 ± 0.28*	1.43 ± 0.32*^ab^

Length (cm)	C	43.14 ± 3.33	42.62 ± 3.22	42.67 ± 3.09	43.07 ± 3.48	43.00 ± 2.57	43.72 ± 2.22	44.70 ± 3.61	46.84 ± 1.86*	47.67 ± 3.93*^a^
	W		42.21 ± 2.88	42.16 ± 3.59	42.34 ± 3.68	43.52 ± 1.54	44.21 ± 3.78	44.38 ± 2.70	45.73 ± 2.14	43.63 ± 2.87^b^
	WS		44.12 ± 3.90	40.63 ± 2.91	43.74 ± 2.49	43.82 ± 2.44	44.29 ± 3.92	44.79 ± 2.46	45.60 ± 3.27	46.11 ± 3.14^ab^

Pectoral fin	C	0.67 ± 0.71	0.33 ± 0.71	1.11 ± 0.60	0.67 ± 0.71^a^	0.67 ± 0.50^a^	0.33 ± 0.50^a^	0.56 ± 0.73^a^	0.33 ± 0.71^a^	0.11 ± 0.33^a^
	W		1.00 ± 0.71	1.56 ± 0.88	0.56 ± 0.53^a^	0.33 ± 0.71^a^	0.22 ± 0.44^a^	0.22 ± 0.44^a^	0.22 ± 0.44^a^	0.56 ± 0.53^a^
	WS		1.00 ± 0.87	1.56 ± 0.73	1.89 ± 0.78*^b^	1.67 ± 0.87*^b^	1.78 ± 0.44*^b^	2.00 ± 0.71*^b^	2.00 ± 0.50*^b^	1.78 ± 0.83*^b^

Pelvic fin	C	0.89 ± 0.60	0.56 ± 0.73	0.67 ± 1.00	0.56 ± 0.53^ab^	0.22 ± 0.44*^ab^	0.11 ± 0.33*^a^	0.44 ± 0.73^a^	0.56 ± 0.88	0.22 ± 0.44*^a^
	W		0.44 ± 0.53	0.89 ± 0.78	0.22 ± 0.44*^a^	0.00 ± 0.00*^a^	0.00 ± 0.00*^a^	0.44 ± 0.53^a^	0.44 ± 0.53	0.33 ± 0.50^a^
	WS		0.67 ± 0.50	1.22 ± 0.67	1.00 ± 0.00^b^	0.78 ± 0.44^b^	0.89 ± 0.60^b^	1.22 ± 0.44^b^	0.78 ± 0.44	1.11 ± 0.60^b^

Anal fin	C	0.67 ± 0.50	0.33 ± 0.50^a^	1.00 ± 1.00	0.67 ± 0.50^a^	0.89 ± 0.60	0.78 ± 0.44^a^	0.67 ± 0.50^a^	0.67 ± 0.50^a^	0.67 ± 0.50^a^
	W		0.78 ± 0.67^ab^	0.67 ± 0.50	0.89 ± 0.60^ab^	1.00 ± 0.71	1.00 ± 0.87^a^	0.78 ± 0.44^a^	0.67 ± 0.50^a^	0.89 ± 0.78^a^
	WS		1.11 ± 0.78^b^	1.22 ± 0.67	1.56 ± 0.53*^b^	1.44 ± 0.53*	1.89 ± 0.78*^b^	1.67 ± 0.71*^b^	2.11 ± 0.60*^b^	1.67 ± 0.71*^b^

Caudal fin	C	1.44 ± 0.88	1.00 ± 0.87^a^	2.00 ± 1.00	1.78 ± 0.67	1.33 ± 0.71	1.44 ± 0.73	1.00 ± 0.87^a^	1.11 ± 0.60	1.00 ± 0.50^a^
	W		1.67 ± 0.50^ab^	2.22 ± 0.44	2.33 ± 0.87*	1.56 ± 0.53	1.78 ± 0.67	1.56 ± 0.53^ab^	1.78 ± 0.67	2.22 ± 0.67^b^
	WS		2.00 ± 0.71^b^	2.22 ± 0.83	2.22 ± 0.67	1.89 ± 0.78	1.89 ± 0.60	2.11 ± 0.33^b^	1.78 ± 0.44	1.67 ± 0.71^ab^

Dorsal fin	C	0.78 ± 0.44	0.22 ± 0.44^a^	0.33 ± 0.71	0.33 ± 0.50	0.56 ± 0.88	0.33 ± 0.50	0.33 ± 0.50	0.11 ± 0.33*^a^	0.11 ± 0.33*
	W		0.56 ± 0.73^ab^	0.33 ± 0.71	0.67 ± 0.50	0.11 ± 0.33*	0.33 ± 0.50	0.33 ± 0.50	0.11 ± 0.33*^a^	0.56 ± 0.73
	WS		0.89 ± 1.05^b^	0.44 ± 0.53	0.56 ± 0.53	0.56 ± 0.53	0.89 ± 0.60	0.78 ± 0.44	0.89 ± 0.78^b^	0.67 ± 0.71

C = Control, W = Wound, WS = Wound and Stress. Values represent means ± SD, n = 9 per treatment/week. Means in a column (week) within the same measurement that have differing superscript letters a-b indicate significant differences (*p* < 0.05). Asterisks* show a significant difference compared to the Pre-stress group (*p* < 0.05).

#### 3.3.1 Fin erosion

Table 3 summarises the average fin score for all experimental groups. Significant differences were found for all five fins when comparing the wound + stress group to the control group and four fins, excluding the caudal fin for the wound group, compared to the wound + stress group. From week 3 until the end of the experiment, there was a significant increase in the erosion of pectoral, pelvic, and fin regions in the wound and stress group compared to the control and wound group. For the wound + stress group, the severity of fin damage ordered from most damaged at week 8 compared to week 1 is pectoral, pelvic, anal, caudal, and dorsal. Figure 4 highlights the positive correlation between the aggregated fin score of the pelvic, pectoral, and anal regions and plasma cortisol in the wound and stress group (rs = 0.66, *p* = 0.085). A weak correlation was shown in the control group (rs = 0.13, *p* = 0.76) and a negative correlation in the wound group (rs = −0.36, *p* = 0.38) groups (Table 3).

### 3.4 Wound healing

**FIGURE 5:**
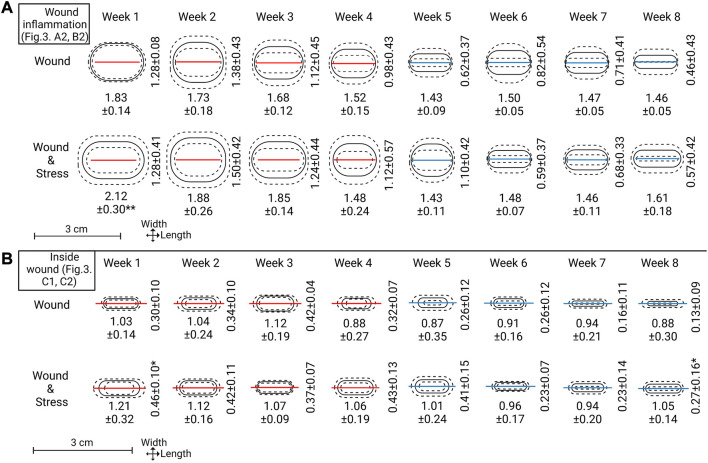
Top section **(A)**: Wound inflammation width and length measurements for each week are represented as means (solid line) ± standard deviation (dashed lines). The red or blue line within the ovals represents the mean length of incision (A1 in Figure 3). A red line indicates not all replicates within that week had wound width (B1 in Figure 3) = 0.0 cm and a blue line indicate all replicates within that week had wound width = 0.0 cm. Lower sections **(B)**: Inside wound healing width and length, represented the same as the top section. Mean incision length lines are carried over to represent scale and show a comparison point to the outside wound (note: section **(B)** is slightly zoomed see the length of scale). Significance between groups for length or width is represented as * = *p* ≤ 0.05.

The visually observed effect of internal tagging and internal tagging with daily stress with a focus on the size of the incision, inflammation, and inside wound was determined by measurements at weekly intervals starting at week one and ending on week 8 (Figure 5, where explanations of A1, A2, B1, B2, C1, C2 can be found in Figure 3 A). The incision length (A1) was not significantly decreased for the two groups and stayed within the 5% range of the original 1.5 cm incision. The width of the incision (B1) for both groups and all individuals at and after week 5 was 0.0 cm, thus, defined as a completely closed wound. Before week 5, the number of fish with completely closed wounds in the wound group compared to the wound + stress group is as follows (n = 9); week 1–0:1, week 2–5:2, week 3–4:3, week 4–6:4, and 9:9 for weeks 5 and onward.

The length of inflammation (A2) gradually declined from weeks 1, 2, and 3 for both groups, and from week 4 onward, it matched the wound incision length. The length of inflammation for the wound + stress group was significantly higher in week 1 (*p* = 0.001) compared to the wound group in week 1. The width of inflammation (B2) gradually decreased from week 2 onward, as both groups had an increase in inflammation width from week 1 to week 2; however, there was no significant difference between the groups at any time. When taking the area of inflammation (A2, B2) and calculating it as an ellipse, the wound + stress group had a significantly larger area of inflammation (*p* < 0.05) in weeks 1, 2, 3, 4, 5 and 8 compared to the wound group while the opposite was true for week 6. No differences between the two groups were seen in week 7.

The inside wound length (C1) gradually decreased in both the wound and wound + stress groups. However, the wound + stress group had higher length values in seven of the 8 weeks. The inside wound width also gradually decreased in both groups and was higher in six of the 8 weeks in the wound + stress group, whereas in weeks 3 and 6, the wound group had higher values. The wound width was significantly higher in week 1 (*p* = 0.003) and 8 (*p* = 0.04) for the wound + stress group compared to the wound group. When taking the area of the inside wound (C1, C2) and calculating it as an ellipse, the wound + stress group had a significantly higher area of inside wounding (*p* < 0.05) in weeks 1, 2, 4, 5, 7, and 8 compared to the wound group while the opposite was true for week 3 and no differences between the two groups were seen at week 6. Additionally, the wound group contained one individual at week 7 with 0 cm inside wound width (healed) and two individuals with 0 cm inside wound width at week 8, while the wound + stress group had no individuals with closed inside wounds during the trial.

## 4 Discussion

While partly overlooked by fisheries biologists, terrestrial biologists and statisticians have given considerable attention to tagging studies to improve techniques that estimate animal population size and mortality (Pine et al., 2003). However, using tags to monitor wild fish has recently become crucial in understanding otherwise hard-to-observe behaviours (Lucas and Baras, 2000; Cooke et al., 2004). Due to the development of aquaculture and technology, using tags to monitor fish has seen increasing interest as providing better welfare through live monitoring can secure healthier animals (Macaulay et al., 2021). While the development of tags proceeds in aquaculture, the effects that the initial insertion causes inflammation and the long-term impact on fish should not be overshadowed. The present experiment suggests that tagged Atlantic salmon post-smolts may not experience chronic stress from the tagging itself. However, tagged fish under daily crowding stress respond with an altered allostatic state and wound healing compared to unstressed fish.

### 4.1 The stress response

Aquaculture-produced fish will encounter stressful situations ranging from mechanical to environmental stressors (Eissa and Wang, 2016). During these stressful events, the fish responds by activating its stress responses, to which the HPI axis is a significant contributor. The HPI axis end product is the release of corticosteroids to redistribute energy utilisation into various organs to combat the altered metabolic demand of stress (Faught et al., 2016). Corticosteroids come in two classes, glucocorticoids (GR) and mineralocorticoids (MR) which can affect metabolism, immunity, and ion regulation (Krasnov et al., 2012; Faught et al., 2016). In a variety of fish species, it has been found that neuroendocrine factors increase rapidly (minutes) after exposure to acute stress and can last for hours. Comparatively, chronic elevations take longer (days/weeks/months) to be visible as controlling and regulating factors within the HPI axis breakdown (Vijayan et al., 2010).

#### 4.1.1 Primary stress responses

CRH (alternatively named CRF; Corticotropin-releasing factor) is a neuropeptide hormone. In fish, it is the main regulatory factor of the stress axis while also having roles in immune response modulation and suppressing appetite, reproduction, and locomotion (Wendelaar Bonga, 1997; Bernier and Peter, 2001; Flik et al., 2006). Chronic stress effects on plasma CRH in fish to our knowledge, is yet to be studied. However, Pepels et al. (2004) showed that acute stress in tilapia caused plasma CRH to peak 11 min after the applied stressor and then decline to pre-stress levels. Findings from Pepels et al. (2004) corroborate similar findings found for humans that suggest plasma CRH has a half-life ranging from 4–20 min (Schürmeyer et al., 1984; Stalla et al., 1986). In mammals, circulating CRH stems from hypothalamic secretion into the hypothalamic-pituitary portal systems. While fish lack this portal vascular system and use direct innervation, it is believed that circulating CRH secretion in fish is associated with the caudal neurosecretory system (CNSS), as well as the lateral part of the ventral telencephalon (brain) and may be produced locally in organs such as the head kidney (Lu et al., 2004; Pepels et al., 2004; Sower, 2015; Gorissen and Flik, 2016).

In the current experiment, a significant increase of CRH within groups occurred during week 3 for the wound + stress group compared to the control group and in weeks 6 and 7 for the control group compared to the wound + stress group. There was, however, no consistent increase within a time point for all individuals within a group. Where increases in plasma CRH in individuals did occur, it did not correlate with any other primary or secondary stress response. Since the crowding stressor in the current experiment was given 24 h before sampling, the peak in circulating CRH produced could not be visible due to its short half-life in the plasma. Interestingly, Pepels et al. (2004) study showed that confinement stress of 48 h eliminated the plasma CRH and cortisol response to a novel acute stressor.

In comparison, the control groups experiencing the same acute stressor showed high plasma CRH and cortisol values. Desensitisation of the HPI axis to a stressor may happen; however, prolonged stressors have been shown to become maladaptive, and this can be seen with the increase of ACTH and cortisol in the latter half of the current study for the wound + stress group but not for CRH (Kristiansen and Bracke, 2020). It has been described that the HPI axis can be activated without CRH in mice *via* CRH-like hormones as long as the CRH receptors are present, and it is also known that fish CRH gene expression in the brain varies greatly between species and types of stressors given (Weninger et al., 1999; Lai et al., 2021). Additionally, it has been described that CRH expression in the brain increases following immune stimulation in goldfish (Volkoff and Peter, 2004). In our study, plasma CRH does not have a similar buildup within the plasma as seen with ACTH and cortisol during the 8-week experiment when sampling is taken 7 days after tagging and 24 h after stressor application.

The significant increase in the wound + stress group for ACTH during weeks 4 and 5, followed by elevated baseline levels of plasma cortisol from week 6, possibly represents HPI-axis changes that may lead to a chronically stressed state described as allostatic overload type 2. To enter this overload state, individuals will have an altered allostatic state that activates primary mediators to help maintain stability through change, where cumulative effects from the allostatic state result in allostatic load. Should allostatic load become a cumulative burden through prolonged exposure to stress, the individual experience allostatic overload. An acute adaptive response (allostatic overload type 1) is initiated when the energy needed exceeds the energy available. Therefore, the release of glucocorticoids causes a decrease in the energy demand of an individual by avoiding normal life history stages, decreasing the allostatic load. When energy needed does not exceed energy available, a chronic non-adaptive response (allostatic overload type 2) is present that increases the level of glucocorticoids, and allostatic load is not reduced (McEwen, 1998; McEwen and Wingfield, 2003; Goymann and Wingfield, 2004; McEwen and Wingfield, 2010). If allostatic overload continues, this results in damage instead of protection to the individual (McEwen and Wingfield, 2010). Baseline plasma cortisol levels in previously unstressed fish can be as low as 13.8 nM, while chronically stressed fish show values above 27.5 nM (Pickering and Pottinger, 1989; Van Zwol et al., 2012; Iversen and Eliassen, 2014). The applied stress used in the current study yielded chronically stressed values ranging up to a mean of 93 nM in week eight. Repeated acute stress has been shown to have higher cortisol values compared to permanent chronic stress. The sampling is taken 24 h after the stressor allowing for a chronic accumulative response to be monitored (Tort et al., 2004). The entering into this chronic stress state is supported not only by primary stress response parameters but also by the increase in the wound + stress groups fin erosion, wound inflammation, and internal wound healing, as they were seen to be significantly increased compared to the wound and control groups at some time points. Chronic stress and the prolonged increase in cortisol have been associated with several tertiary stress responses such as; decreased growth rates (Sadoul and Vijayan, 2016), reproductive dysfunction (Schreck, 2010; Pankhurst, 2016), increased susceptibility to disease (Yada and Tort, 2016), decreased survival (Gomes et al., 2003; Schreck and Tort, 2016), and disruption in osmoregulation (Sampaio and Freire, 2016; Vargas-Chacoff et al., 2021).

At all levels of the HPI-axis, the release of cortisol due to stress is regulated by negative feedback and inducing and inhibiting factors (Wendelaar Bonga, 1997; Mommsen et al., 1999). The negative feedback regulation in the wound + stress group possibly functioned from week one to three, but after that, the cortisol within the wound + stress group continued to increase gradually. A gradual decrease in HPI-axis reactivity has been previously observed in chronically stressed fish, where habituation and resistance to stress occur (Madaro et al., 2016; Moltesen et al., 2016). In the current study, habituation is not seen. It can be attributed to either a high stressor level or length of the experiment where ending the investigation too early might result in the conclusion of habituation, as could be assumed to be the case for the first half of this study. While the negative feedback regulation becomes dysregulated, its effect on dopamine is shown by lower levels in the stress + wound group compared to the wound group. Brain monoaminergic systems have been shown to increase dopaminergic activity, however, the available data is limited, and data for plasma dopamine regarding chronic fish stress is undocumented (Weber et al., 2012; Gesto et al., 2013). However, it has been shown that within the brain, dopamine activity is reduced by pro-inflammatory cytokines in Senegalese sole (Weber et al., 2015). In the current study, plasma dopamine was highest in all groups at week eight, where the holding tanks contained the lowest number of fish. If confirmed, increasing plasma dopamine by reducing tank density could impact study outcomes not currently considered in experimental designs.

When considering internal tagging, the implantation process for the wound group was shown to cause no chronic stress when the first sampling was taken 7 days post-wounding. Additionally, inflammation during wound healing did not activate the stress response; therein, no detectable bidirectional communication between the HPI-axis and the immune system was seen regarding the wound group within the framework of the study. However, bi-directional communication with interactions between the immune-and endocrine network, cannot be dismissed as a possible influence on the chronic stress response in the wound + stress group through elevated stress hormones, higher inflammation and slower wound healing in inside wounds (Stolte et al., 2008; Pérez-Casanova et al., 2010; Tort, 2011; Wendelaar Bonga, 2011; Tort and Balasch, 2022). The introduction of a wound is followed by an inflammation response which will produce inflammatory cytokines (discussed in 4.2) that are under glucocorticoid control. Yet the exact role of cytokines within the stress axes is ill-defined (Tort and Balasch, 2022). What has been described is mainly the up and downregulation of pro and anti-inflammatory cytokines by glucocorticoids in various fish species (Verburg-van Kemenade et al., 2011; Nardocci et al., 2014; Philip and Vijayan, 2015; Yarahmadi et al., 2016; Khansari et al., 2017b; Reyes-López et al., 2018). The increase in cytokines following inflammation has the potential to activate the stress response, as is apparent with interleukin-6, where it has a role as a stimulating factor of CRH, prolactin, growth hormones, and ACTH, which in turn will increase cortisol (Calcagni and Elenkov, 2006; Žarković et al., 2008). Therefore, although the wound group showed no increase in stress responses, the cumulative effect of additional load on the HPI-axis through a daily stressor and the possible increase in cytokines should be considered.

#### 4.1.2 Secondary stress responses

Secondary stress responses are reactive changes within the individual’s physiology depending on the stressor it has encountered and is affected by primary stress responses. The most commonly measured secondary stress responses include; (1) glucose which is influenced by increased catabolism as well as hormonal-stimulated glycogenolysis and gluconeogenesis; (2) lactate which fluctuates due to muscle glycolysis that produces ATP through anaerobic metabolism; and (3) ionic or osmolality changes resulting from the increase in catecholamine and cortisol release (Mommsen et al., 1999; Weber and Shanghavi, 2000; Liebert and Schreck, 2006; McEwen and Wingfield, 2010; Pankhurst, 2011; Sopinka et al., 2016; Weber et al., 2016; Noble et al., 2018). The effects of chronic stress on the secondary stress responses occurred during week eight, as magnesium, calcium, chloride, and osmolality were dysregulated in the wound + stress group. Additionally, the wound + stress group had significantly higher chloride levels in weeks 1, 3, 4, 5, and 8 compared to the wound group. This shows chronic stress and the later increase of cortisol through the breakdown of the HPI-axis *via* over-sensitivity of ACTH. However, this had few effects on secondary stress responses in this study. The stress + wound group showed changes in all four ionic parameters at week eight, which could signify the start of the breakdown of the osmoregulatory systems. Catecholamines have been shown to affect gill permeability and thus can disrupt the regulation of internal ions (Barton et al., 2003).

With the sampling protocol used, one can conclude that surgery does not cause a long-term increase in secondary stress responses and that the instigation of chronic stress in the wound + stress group does not seem to affect the secondary stress responses severely. However, care should be taken in being too conclusive, as the onset of changes in the HPI-axis only manifested itself after week 6 in the wound + stress group, as plasma cortisol became elevated 2 weeks before the end of the experiment. Notably, Iversen and Eliassen (2014) and Patel et al. (2022) previously showed that a group of Atlantic salmon and lumpsucker (*Cyclopterus lumpus L*.) that entered allostatic overload type 2 created only pronounced changes in plasma magnesium while other secondary responses (glucose, lactate, osmolality, chloride) were unaffected by the altered state of the HPI-axis during a six to 8-week trial.

Understanding the time frames during active stress responses should be considered. Experimental design and measurement times can describe different situations as the recovery of primary and secondary stress responses are fast (minutes to hours) after acute stress, while the effects of chronic stress can take weeks to develop, as seen in the current study (McDonald and Milligan, 1997; Wendelaar Bonga, 1997; Mommsen et al., 1999). Concerning animal welfare, it is imperative to remember that severe acute and prolonged chronic stressors can be shown not to affect the fish at a specific time when they are out of the peak zones and when studies are not carried out with long enough duration to develop the negative impacts. With this in mind, we acknowledge that the current study only focuses on chronic buildups and can miss any acute issues arising immediately after stress or those regulated before the sampling 24 h after applied stress.

#### 4.1.3 Tertiary stress responses

##### 4.1.3.1 Weight and length

When a prolonged stressor threatens homeostasis, the organism must distribute energy to the most vital processes to survive the stressor and neglect energy input into processes such as growth and reproduction due to the energetic cost of initiating the stress response (Wendelaar Bonga, 1997; Ashley, 2007; Wendelaar Bonga, 2011). In the current study, all groups showed a steady rise in growth. While stress and reduced growth rates are commonly associated, no significant growth reduction in the wound + stress group suggests that energy partitioning and inhibition of muscle growth promoters did not substantially occur (Faught et al., 2016; Sadoul and Vijayan, 2016).

Research into salmonid wound healing where stress is not considered has shown no difference between wounded groups compared to control groups in growth (Jensen et al., 2015; Liss et al., 2016; Schmidt et al., 2016; Sveen et al., 2018). When stress is present in the absence of wounds, it is shown to slow growth, likely from moving towards structural protein breakdown and inhibiting myogenesis and hormonal growth regulation, such as the impact cortisol has on the growth hormone/insulin-like growth factor I system (Sadoul and Vijayan, 2016; Vargas-Chacoff et al., 2021). Sveen et al. (2018) showed that chronically stressed Atlantic salmon with wounds did not significantly differ in body weight compared to control fish and that the control fish were slightly larger at the end of the experiment (57 days); these results are similar to our findings. This leads to the possible theorisation that wounded fish under stress cannot completely shift energy out of growth and reduce cytokine signalling, which happens when wounds are not present (Philip and Vijayan, 2015). This may be due to the need for growth factors and cytokines in the wound-healing process. Severe wounds allow the external environment to contact the internal, which can ultimately lead to death. As such, it is essential to allow repair to occur for survival. However, the current study found that internal wounds heal slower in stressed than in non-stressed individuals. While data to support this theory of wounds interfering with growth suppression through stress is lacking, it is interesting to find other systems where overall adverse effects can cause minor beneficial effects. One such system is the immune system which can benefit from otherwise debilitating factors, where acute stress, to some extent, has short-term positive effects on immunity compared to chronic effects (Khansari et al., 2017a; Khansari et al., 2017c). Had the current study run more extended, a decrease in growth could have been seen, as the HPI-axis broke down in the later part of the study, and wounds began to be completely healed. Having a group of only stressed fish without wounds and a group stressed before wounding could have helped to clarify some more.

##### 4.1.3.2 Fin erosion

Fin erosion can be defined as the erosion of the epidermis, dermis, and fin rays which can be seen as damage to the fins in the form of fraying, splitting, size reduction, and loss of standard shape (Latremouille, 2003; Ellis et al., 2008). Fin erosion has been seen extensively with the onset of aquaculture, as it is found much less in the natural environment. Thus, due to its direct association with aquaculture and being externally visible, it has been accepted as a meaningful operational welfare indicator in fish (Ellis et al., 2008; Stien et al., 2013; Noble et al., 2018). Fins of fish are nociceptive, contain nerve cells, and have been shown by Roques et al. (2010) to have an acute response to fin clipping. This highlights the seriousness of fin erosion when discussing fish welfare. Fin erosion has been shown to have the capacity to heal, even to the extent that whole fins can regenerate, as seen in Zebrafish, suggesting that under good welfare conditions, fins should heal even after stressful events (Ellis et al., 2008; Pfefferli and Jaźwińska, 2015).

The experiment shows an increase in fin damage which appeared to be generally higher for all fins in the wound + stress group and had a significant increase towards the latter half of the experiment, starting at week three for the pectoral, pelvic, and anal fins when compared to the control and wound groups. This pattern is faintly present in the caudal and dorsal fins. When averaging the pectoral, pelvic and anal fins together, a positive correlation between cortisol and fin erosion was seen in the wound + stress group compared to the control and wound group. A positive correlation between fin erosion and scale cortisol has been described previously in rainbow trout (Weirup et al., 2021). Gregory and Wood (1999) also showed that cortisol-injected rainbow trout had significantly higher fin erosion than untreated fish. The current study shows that a daily chronic crowding stressor lasting 30 min causes fin erosion with differing rates and severity on each fin. While the most damaged fins appear on the ventral surface and the stressor applied was to crowd the fish, it can be assumed that tank and conspecific abrasion had a role in increased fin erosion along with aggressive behaviour (Noble et al., 2012). Interestingly, fin erosion is not seen in all species of farmed fish, leading to theorising that species with similar rearing practices and morphology must sustain damage from behavioural differences such as the absence or presence of aggressive fin nipping (Ellis et al., 2008). In future studies, a different stress method, such as chasing, can be used to assess how much impact tank abrasion had on fin erosion.

Fin erosions aetiology is not fully understood and is mainly theorised to be from abrasion with surfaces, aggressive behaviour in the form of nipping and biting, poor water quality, feeding regime, and in some cases, increased density (Ellis et al., 2008; Jones et al., 2010). Fin erosion can be viewed as a stressor that activates the HPI axis, which in turn will release glucocorticoids which have been shown to disturb the routine healing of wounds (Sveen et al., 2019). Øverli et al. (2002) demonstrated that 48-h cortisol treatment inhibited aggressive behaviour in rainbow trout, while 1-h short-term treatment did not inhibit nor significantly increase aggression. This may suggest that initial fin erosion can be caused by aggressive behaviour in stressed fish due to the resulting acts of causing the stressor, while prolonged erosion may be due to abnormal healing of fins by sustained HPI-axis activation or a possible culmination of both working simultaneously. Understanding the effects and relationships that cause fin erosion on each specific fin with biological factors, such as cortisol, or farming factors, such as feeding practices, can significantly increase our capacity to respond to harmful effects on welfare. The ease with which one can assess fin erosion should be appealing, as it can be a complementary observation to mandatory handling processes as well as possibly measured non-invasively with emerging imaging technology used for salmon lice counting (Cvetkovikj et al., 2015; He et al., 2016; Guragain et al., 2021).

### 4.2 Wound healing and tagging

When considering tagging fish, the main concerns are the effects on welfare, the healing process, survival rate, and retention of tags. Using a surgical tagging procedure and manipulating wounds by sutures, the current study provides insight into how healing proceeds in a controlled environment and how a daily crowding stressor can influence the visible healing process on the outside and inside of Atlantic salmon skin. One observed more significant inflammation area of wounds and slower wound healing closure on the inside for the wound + stress group compared to the wound group. Stress has slowed wound healing in humans, mice, reptiles, and several fish species (Kiecolt-Glaser et al., 1995; Marucha et al., 1998; Padgett et al., 1998; French et al., 2006; Sveen et al., 2018). A comprehensive review of stress and the immune system in fish has been established by Tort and Balasch (2022), while assessments for stress and wound healing have been established for humans (Christian et al., 2006; Vileikyte, 2007). The effect of chronic stress on deep cutaneous wounds in Atlantic salmon has been described by Sveen et al. (2018), as well as the impact of hydrocortisone implants on wound repair described by Roubal and Bullock (1988). The current study also confirms the expectation of wound healing slowing when the fish are chronically stressed.

The immune system and stress system have a location of significant crosstalk in the head kidney, where chromaffin cells, interrenal cells, and hematopoietic tissue are located to form a system known as the neuroimmunoendocrine regulatory feedback system (Tort and Balasch, 2022). The effects of stress on healing are mainly through the influence glucocorticoids have on pro-inflammatory cytokines such as interleukin-1β (IL-1β), IL-1α, IL-6, IL-8, and tumour necrosis factor-α (Christian et al., 2006; Guo and DiPietro, 2010; Serra et al., 2017; Tort and Balasch, 2022). In the current study, an increased inflammation exaggerated in the first 2 weeks for the stress + wound group can be identified compared to the wound group. Similar results to the current study of increased inflammation are presented by Sveen et al. (2018), who concluded that high fish density increased the transcription levels of inflammatory genes in Atlantic salmon within the first-week post wounding in the wound site. However, Hou et al. (2019) showed that in unwounded stressed rainbow trout, cortisol inhibits the release of pro-inflammatory cytokines IL-1β and TNF-1α, where slight activation of the HPI-axis can increase anti-inflammatory cytokines. There is a difference in the effect acute and chronic stress have on the immune system, and while cortisol regulation broke down at week 6 in the current study, inflammation in cold-water species of fish is most severe in the first 2 weeks (Tort, 2011; Sveen et al., 2020). Therefore, the inflammation occurs under daily stress where the chronic breakdown has not happened yet. In mice, an acute stressor given 24 h before another acute stressor showed accelerated cytokine production. Yet, the amplitude of increase was unaffected compared with only acutely stressed mice (Cheng et al., 2015). Thus, pro-inflammatory cytokines are released as a normal part of the inflammation process in wounds, and stress increases the time for wounds to heal. Stress can also reduce and increase the expression of specific pro-inflammatory cytokines, where concurrent stress possibly increases the rate of release post-stress but not amplitude (Johnson et al., 2005; Kiecolt-Glaser et al., 2005; Christian et al., 2006; Vileikyte, 2007; Tort, 2011; Manikowska et al., 2014; Cheng et al., 2015).

Despite its shortcomings, inside wound healing presents a unique method to isolate wound healing to muscular healing with an internal medium where the surface is scaleless, consists of no outer mucus and is free from abrasion that might be experienced during studies. In the inside, wound healing progressed slower for fish suffering from stress. They also presented zero completely healed wounds at the end of the experiment, whereas the wound-only group contained two fish with completely healed inside wounds at week eight. Inside wound healing times are often unconsidered and are vital when considering tag retention, as various internal tagging studies on multiple species have shown varying levels of tag retention (Hadden et al., 2018; Gerber et al., 2019; Walton-Rabideau et al., 2019; Macaulay et al., 2021; Marsden et al., 2021; Matthew, 2021). The current study highlights the importance of placing internal tags away from the incision site as much as possible to eliminate the protrusion of unhealed internal wounds by tags. The current study also highlights that the incision held by sutures closed on the surface within 4 weeks at 7.3 ± 0.3°C in stressed and unstressed Atlantic salmon. As Deters et al. (2012) suggested, suture retention is only beneficial up to the point of complete healing. After that, it becomes a point of increased inflammation and a source of infection. Thus, as telemetry tagging becomes more common in aquaculture, tagged fish should be maintained as stress-free as possible to ensure well-healed wounds and to identify sutures that can hold retention up to the point of healing. The measurement of internal healing times for target species should be considered and conducted as increased understanding can increase the success of tag retention, wound healing, survival, and ultimately the welfare of the individuals. While this study offers insight into tagging previously unstressed fish, this can be an unrealistic goal to achieve in the grow-out phase of aquaculture. Therefore, tagging fish subject to previous acute and chronic stress will allow further insight into possible harmful effects on wound healing and welfare and can help to contribute to the successful use of tags.

## 5 Conclusion

The main observations from the study are: (1) Wounded individuals do not suffer from chronic stress or chronic adverse welfare effects; (2) Wound + stressed individuals suffer from allostatic overload type 2 with an increase of ACTH starting at week four to an increase in baseline levels of plasma cortisol starting at week six; (3) Fin erosion is significant in stressed fish where damage to fins occurs well before the allostatic overload, indicating the possible use of fin erosion as a pre-indicator of chronically stressful conditions; (4) Stressed individuals suffer a more extensive inflammation period in weeks 1-2 while their inside wounds heal slower than unstressed wounded fish; (5) Due to the outside wound being sealed at week 4, the use of absorbable sutures should be considered with 4 weeks retention times to improve welfare and reduce unwanted damage; (6) Tag retention can be improved by placing tags away from the site of incision as internal wound healing was seen taking place in weeks 7 and 8 for a few unstressed individuals. Altogether, the results indicate that internal tagging, assuming a stress-free environment, does not compromise welfare within this study’s selected parameters. While on the other hand, chronic stress disrupts the healing process and dysregulates the HPI axis, compromising welfare. Thus, tagging in grow-out facilities should prepare for a period of minimized stress post-tagging to provide the best welfare and wound healing.

## Data Availability

The raw data supporting the conclusions of this article will be made available by the authors, without undue reservation.
